# Clinical outcomes of dogs with high-grade cutaneous mast cell tumors

**DOI:** 10.3389/fvets.2024.1519636

**Published:** 2025-01-21

**Authors:** Siew Mei Ong, Charly McKenna, Christopher Pinard, Danielle Richardson, Michelle L. Oblak

**Affiliations:** ^1^Department of Companion Animal Medicine and Surgery, Faculty of Veterinary Medicine, Universiti Putra Malaysia, Serdang, Malaysia; ^2^UPM-MAKNA Cancer Research Laboratory, Institute of Bioscience, Universiti Putra Malaysia, Serdang, Malaysia; ^3^Department of Clinical Studies, Ontario Veterinary College, University of Guelph, Guelph, ON, Canada; ^4^Department of Oncology, Lakeshore Animal Health Partners, Mississauga, ON, Canada; ^5^Centre for Advancing Responsible & Ethical Artificial Intelligence, University of Guelph, Guelph, ON, Canada; ^6^ANI.ML Research, ANI.ML Health Inc., Toronto, ON, Canada

**Keywords:** canine, high-grade cutaneous mast cell tumor, surgical excision, lymphadenectomy, adjuvant therapy

## Abstract

**Objectives:**

To evaluate the prognostic factors and treatment outcomes in dogs with high-grade cutaneous mast cell tumors (HGMCTs).

**Methods:**

Medical records of dogs with a histopathologic diagnosis of HGMCTs were reviewed from a single institution. Clinical factors, treatment-related variables, and adjuvant therapies were documented to evaluate their association with clinical outcomes. Comparative and survival analyses were conducted using Kaplan–Meier survival analysis, log-rank, and Fisher’s exact tests.

**Results:**

The overall median survival time for the 77 dogs was 317 days (range 20–3,041 days) with 6-month, 1-year, and 2-year survival rates of 69, 50, and 30%, respectively. Surgically treated dogs had significantly prolonged survival and were 6.88 times more likely to survive beyond 5.5 months. The presence of metastasis at initial staging was strongly associated with poorer outcomes, as dogs without metastasis at initial staging had 6.94 times higher odds of surviving beyond 2 years. Surgical sites with incomplete margins had a higher local recurrence rate (58%) compared to those with clean margins (26%). Despite aggressive treatment, 75% of the dogs that received concurrent surgical and adjuvant therapy experienced disease progression. Lymph node extirpation, tumor localization, number of tumors, and local recurrence were not associated with the overall outcome.

**Clinical relevance:**

The combination of aggressive local therapy and adjuvant systemic chemotherapy provides a notable survival benefit in dogs with HGMCTs. The limited therapeutic benefit of locoregional lymph node extirpation, combined with a persistently high metastatic rate despite systemic chemotherapy, highlights the critical need for more effective regional and systemic treatment approaches for HGMCT patients.

## Introduction

Mast cells are granular immune cells widely recognized for their central role in various inflammatory and immunological reactions (1, 2). These cells derive from hematopoietic stem cells and migrate to various tissues, particularly surfaces exposed to the external environment, such as the skin, respiratory tract, and gastrointestinal tract (1, 2). The mechanisms behind the neoplastic transformation of mast cells remain largely unidentified; however, underlying genetic causes and KIT mutations have been implicated (3–6).

Mast cell tumors are the most common cutaneous malignancy in dogs, exhibiting significant variation in presentation and biological behavior, ranging from benign to highly aggressive forms with markedly greater metastatic potential (7–11). Furthermore, the manifestation of paraneoplastic disorders, attributable to the release of histamine, heparin, eosinophil chemotactic factor, and proteolytic enzymes from mast cell granules, including Darier’s sign, gastrointestinal ulceration, coagulopathy, hypotension, and circulatory collapse, presents additional challenges in managing these tumors (12–14). Significant attention has been paid to the search for prognostic markers to guide treatment decisions, with factors such as histologic grade (10, 11, 15–17), mitotic count (18), clinical stage (19, 20), anatomic location (21), microvessel density (22), and c-kit gene mutations (6, 23, 24) being explored.

Histologic grade is generally considered the most reliable and consistently predictive factor for canine cutaneous mast cell tumors (MCTs) (10, 11, 16, 20). The 3-tier grading scheme (Patnaik), widely adopted since 1984, classifies cutaneous MCTs into either grade I (low-grade), II (intermediate-grade), or III (high-grade) (10), while a newly proposed 2-tier grading scheme (Kiupel) divides them into low and high grades (16). Most low-grade cutaneous mast cell tumors (LGMCTs) are effectively treated with wide surgical excision alone, though a small subset of LGMCTs may exhibit aggressive behavior, leading to metastasis and potentially death (25). In contrast, dogs with high-grade tumors have a poorer prognosis, with metastatic rates ranging from 55 to 96%, and deaths often occurring within the first year after diagnosis (10, 11, 16, 17, 20, 26). In recent years, numerous clinical studies have been conducted to evaluate the behavior and therapeutic strategies for HGMCTs, including neoadjuvant vinblastine administration (27), combination therapy with vinblastine and toceranib phosphate (28), lomustine and prednisone therapy (29), and lymph node extirpation (30). However, most were non-randomized trials with inadequately small sample sizes, likely due to the low incidence of HGMCTs (4–20%) and complexity of randomized controlled trials (10, 16, 31, 32).

The aim of this retrospective study was to expand our current understanding of the prognostic factors and outcomes in dogs with HGMCTs treated with different therapeutic protocols in the clinical setting.

## Methods

Medical records from client-owned dogs presented to the Ontario Veterinary College Companion Animal Hospital, University of Guelph, were retrospectively reviewed for histopathologic diagnoses of high-grade (Kiupel), grade II (Patnaik) with histologic criteria consistent with Kiupel high-grade, or grade III cutaneous MCTs between 2007 and 2024. Only dogs with a follow-up period of at least 6 months or those that died within 6 months of an HGMCT diagnosis were included in the analysis. Dogs with multiple MCTs, at least one which was HGMCT, and those diagnosed with HGMCT post-mortem who met the inclusion criteria were also included in the analysis. Dogs with mucocutaneous or subcutaneous tumors were excluded from this study.

Data collected from the medical records included patient signalment (age, breed, sex, and body weight), diagnostic and initial staging investigations, treatment details (date of incisional or excisional biopsy, completeness of surgical excision, lymphadenectomy), as well as information on chemotherapy and radiation therapy, if administered. Post-mortem findings, when available, were also recorded. Follow-up information was retrieved from medical records or obtained through telephone communication with referring veterinarians.

Tumor characteristics evaluated included anatomic location (head/neck, trunk, limb, perineum/inguinal/prepuce/tail, or multifocal if tumors were located at more than one of these sites) and whether there was a history of previous MCT. Surgical excision was considered complete if no microscopic residual tumor was present at the resection margin, and the presence of residual tumor at the resection margin was considered an incomplete excision. Local recurrence was defined as regrowth of a tumor at the surgical site or as indicated in the medical records, and tumors that developed at distant sites following treatment initiation were classified as *de novo* lesions. The location of metastatic disease was recorded based on cytologic or histologic findings during restaging procedures or post-mortem examination.

Median survival time was calculated from the date of treatment initiation (either surgery or neoadjuvant chemotherapy) to the date of death or censoring. Dogs were censored if they were lost to follow-up, dead due to MCT-unrelated causes, or alive at the time of statistical analysis. Continuous data were analyzed for normality using the Shapiro–Wilk test. Normally distributed data were expressed as mean ± SD, and non-normally distributed data were expressed as median (range). Survival plots were generated using the Kaplan–Meier product-limit method. Variables evaluated for association with MST included sex, anatomic location, presence of metastasis at initial staging, treatment protocol (surgical excision, lymphadenectomy, and administration of chemotherapy with or without radiotherapy [RT]), and completeness of surgical excision. Associations between various categorical variables and outcomes (surgical margin, local recurrence, and metastasis) were evaluated using Fisher’s exact test. All statistical analyses were performed using SPSS Statistics version 29.0 (IBM Corp., Armonk, NY, USA), and *p*-values of <0.05 were considered significant.

## Results

Seventy-seven client-owned dogs were included in this study. The mean age at diagnosis was 8.3 ± 0.3 years (range 2.0–13.8 years), and the median weight was 28.5 kg (range 3.4–83.0 kg). The study population consisted of 39 spayed females, 1 intact female, 34 castrated males, and 3 intact males. Twenty-six breeds were identified; Labrador Retrievers (*n* = 17, 22%), mixed breeds (*n* = 17, 22%), and Golden Retrievers (*n* = 7, 9%) were over-represented. With six retriever mixes included in the mixed breed category, retrievers accounted for up to 39% (*n* = 30) of the study population. Bulldog-related breeds – such as Boston Terrier (*n* = 3), American Bulldog (*n* = 2), French Bulldog (*n* = 2), English Bulldog (*n* = 1), and Alapaha Blue Blood Bulldog (*n* = 1), comprised 12%.

The primary HGMCT location was recorded as trunk (*n* = 28, 36%), limb (*n* = 20, 26%), head/neck (*n* = 19, 25%), perineum/inguinal/tail/prepuce (*n* = 7, 9%), and multifocal (*n* = 3, 4%). Staging investigations were not standardized but included thoracic radiographs (*n* = 59, 77%), abdominal ultrasound (*n* = 67, 87%), cytologic and/or histologic evaluation of regional lymph nodes (*n* = 32, 41%), cytology of the liver (*n* = 46, 60%), spleen (*n* = 50, 65%), and bone marrow (*n* = 2, 0.03%), and imaging via computed tomography (*n* = 2, 0.03%) and magnetic resonance (*n* = 1, 0.01%). Metastasis was identified in 25 dogs (37%), including 22 with regional lymph node involvement, two with metastasis to both the lymph nodes and spleen, and one with dissemination to the lymph nodes, spleen and liver. Additionally, 23 dogs (30%) with HGMCT also had LGMCT or subcutaneous MCT. Dogs underwent various non-standardized restaging procedures at clinician or owner discretion, including thoracic radiographs, abdominal ultrasound, and cytologic examination of the liver, spleen, regional lymph nodes, and new tumors every 3 or 6 months. Post-mortem examinations (PM) were conducted on eight dogs. At the end of the study, metastasis was documented in 58% of the study population (*n* = 45). The most commonly affected sites were the lymph nodes (*n* = 27, with 6 confirmed through PM), spleen (*n* = 14, with 4 confirmed through PM), and liver (*n* = 10, with 5 confirmed through PM). Less frequently affected sites, all identified through PM, included the kidneys (*n* = 3), lungs (*n* = 2), heart (*n* = 2), and bone marrow (*n* = 2). Single occurrences were observed in the adrenal gland, pleura, pancreas, gastrointestinal tract, omentum, and mesentery.

**Figure 1:**
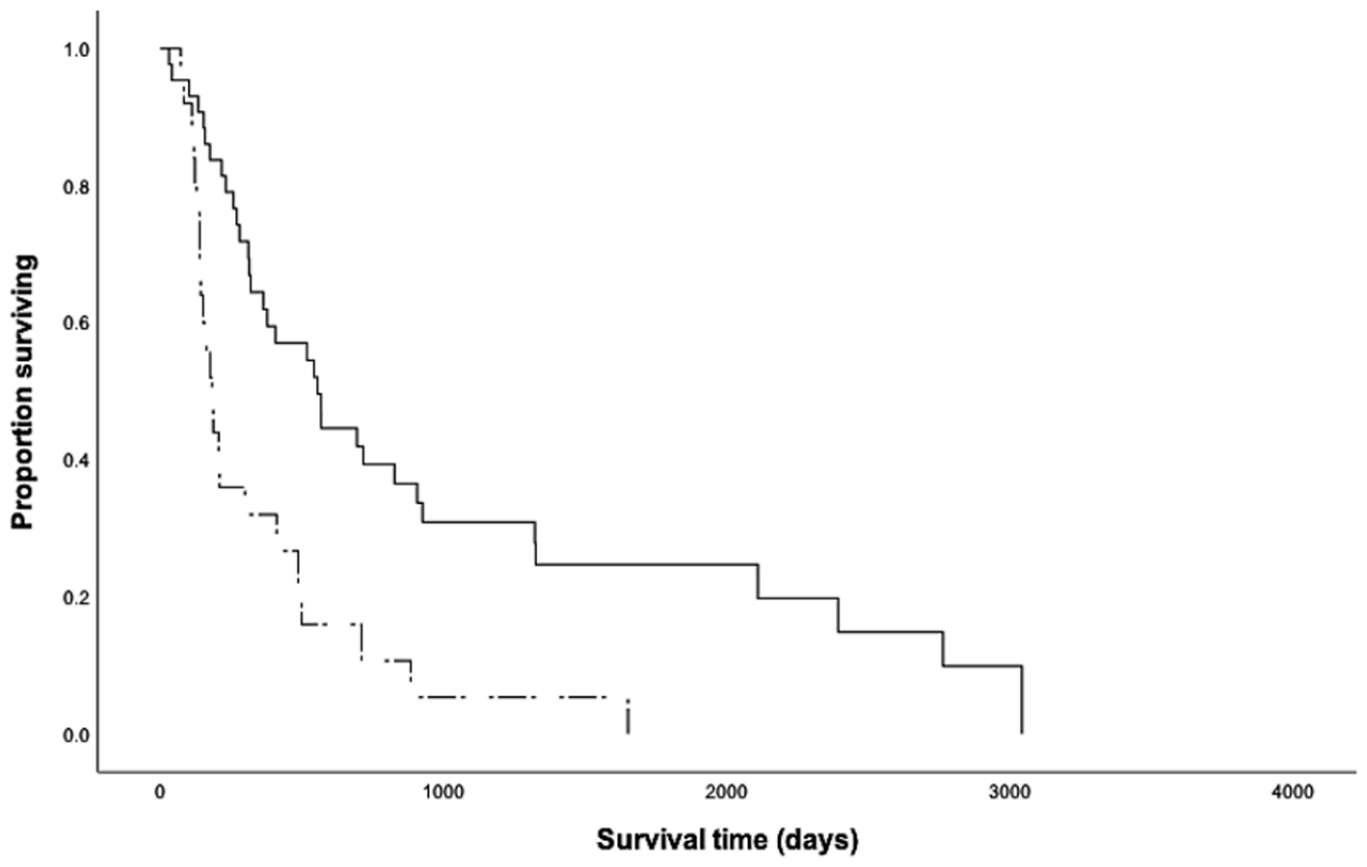
Kaplan Meier survival curves for 68 dogs with high-grade cutaneous mast cell tumors, with (*n* = 25, dashed line) and without (*n* = 43, solid line) metastasis at initial staging. The median survival time of dogs without metastasis (554 days) was significantly longer than that of dogs with metastasis at initial staging (182 days, *p* < 0.001).

The overall median survival time (MST) was 317 days (range 20–3,041 days), with 6-month, 1-year, and 2-year survival rates of 69, 50, and 30%, respectively. The primary tumor location of the HGMCTs, and presence of additional LGMCT or subcutaneous MCT did not have a significant impact on metastasis or MST. However, dogs presenting with metastasis at initial staging had a significantly shorter MST (*p* < 0.001, MST = 182 days, Figure 1). Notably, dogs without metastasis at initial staging had 6.94 times higher odds of surviving beyond 2 years.

**Figure 2:**
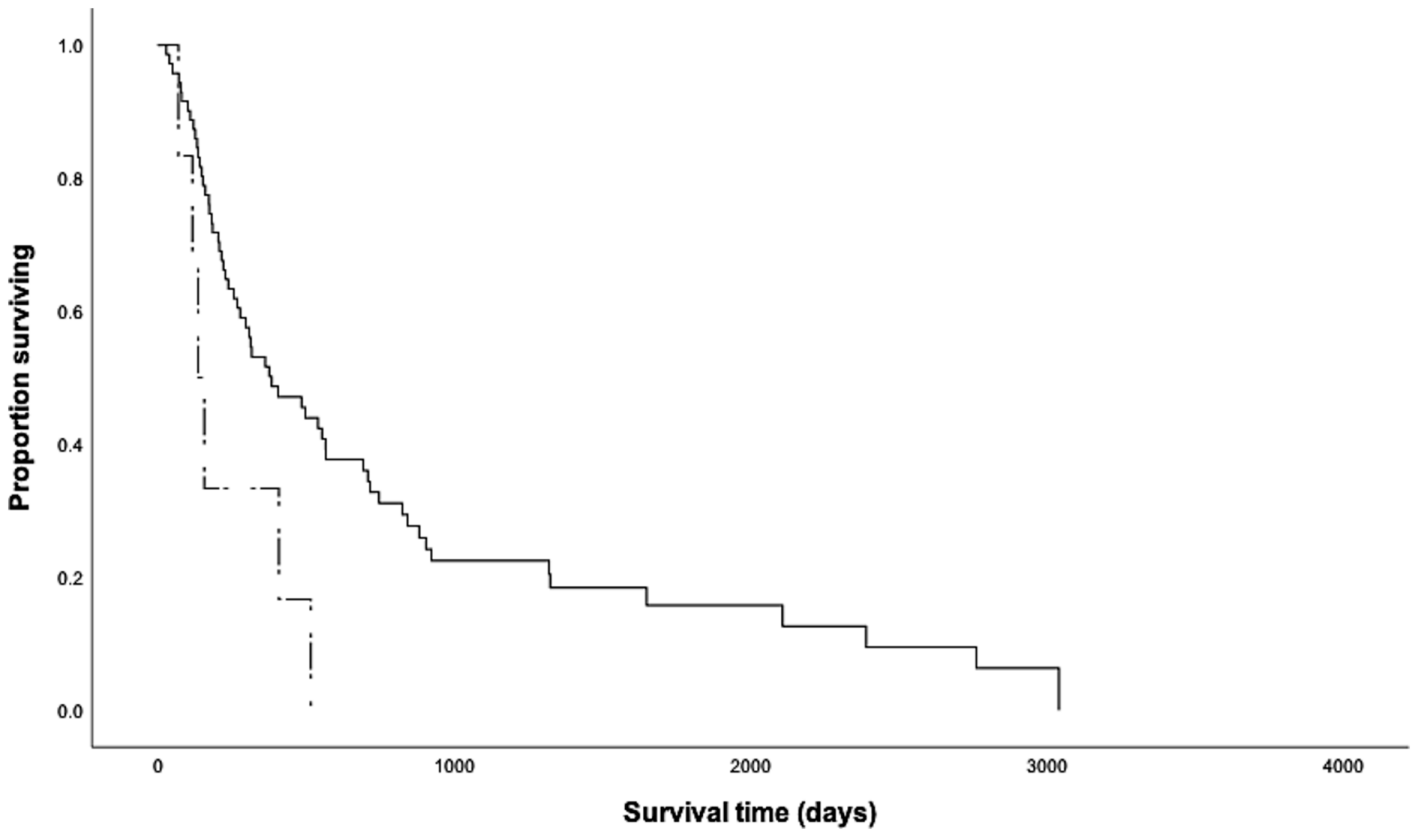
Kaplan Meier survival curves for 77 dogs with high-grade cutaneous mast cell tumors that underwent (*n* = 71, solid line) and did not undergo (*n* = 6, dashed line) surgical excision of the tumors. The median survival time of dogs that underwent surgery (385 days) was significantly longer than that of dogs that did not undergo surgical excision (137 days, *p* = 0.016).

Of the cohort, 65 dogs received chemotherapy following tumor resection, six dogs underwent surgical treatment only, five dogs were treated with chemotherapy with or without RT, and one dog did not receive any treatment based on clinician or owner discretion. First-line and salvage chemotherapies included vinblastine-based protocols, CCNU-based protocols, tyrosine kinase inhibitors (toceranib, imatinib or masitinib), vincristine with cyclophosphamide, intralesional triamcinolone injection, hydroxyurea, and prednisone. The MST of dogs that underwent surgical treatment (*n* = 71, MST = 385 days) was longer than that of dogs that received non-surgical treatment only (*n* = 6, MST = 137 days, *p* = 0.016, Figure 2). Dogs that underwent surgical treatment had 6.88-fold higher odds of surviving beyond 5.5 months. Despite aggressive treatment, 75% (*n* = 49) of the dogs that received concurrent surgical and non-surgical therapy (*n* = 65) experienced disease progression, including *de novo* lesions (*n* = 32, 49%), local recurrence (*n* = 17, 26%) and metastases to the lymph nodes (*n* = 24, 37%), spleen (*n* = 12, 18%) and liver (*n* = 8, 12%). Although not statistically significant, surgical patients who received combined chemotherapy and RT (*n* = 17, MST = 716 days) showed better survival outcomes than those who received chemotherapy alone (*n* = 48, MST = 317 days, Table 1). The dog, which did not undergo any treatment, survived for 409 days before humane euthanasia was performed due to disease progression.

**Table 1:** Univariate analysis evaluating the influence of selected clinical factors on survival in dogs with high-grade cutaneous mast cell tumors.

Variable	*n*	6-month survival (%)	1-year survival (%)	2-year survival (%)	MST (days)	*p* value
Location						
Trunk	28	71	43	36	376	0.204
Limb	20	75	65	27	409	
Head/neck	19	74	53	27	309	
Perineum/inguinal/ tail/prepuce	7	57	57	38	567	
Multifocal	3	33	0	0	157	
Metastasis at initial staging						
No	43	83	62	39	554	<0.001
Yes	25	48	32	11	182	
Surgical treatment						
No	6	33	33	0	137	0.016
Yes	71	73	52	33	385	
Surgical treatment with						
No chemotherapy/RT	6	33	33	33	110	0.268
Chemotherapy	48	73	48	28	317	
Chemotherapy + RT	17	88	69	46	716	
Lymphadenectomy						
No	50	70	54	36	406	0.112
Yes	27	70	44	20	309	
Histopathological margin						
Complete	33	97	72	58	882	0.108
Incomplete	16	94	68	14	517	

RT = Radiation therapy.

Lymphadenectomy was performed in 27 dogs (35%), with histopathologic analysis available for 25 of them. Lymph node metastasis was detected in 63% (*n* = 17) of the excised nodes. Lymph node extirpation did not result in a significant survival advantage (Table 1), nor did it lead to a notable reduction in metastatic spread. Moreover, lymphadenectomy did not provide a clear survival benefit for dogs with metastatic lymph nodes (*p* = 0.694). The MST for dogs with metastatic lymph node that did not undergo lymphadenectomy was 161 days, compared to 182 days for those that underwent the procedure.

To assess the impact of surgical margins on local recurrence, only surgical sites with a minimum follow-up period of 6 months postoperatively were analyzed, unless recurrence occurred within 6 months. A total of 54 HGMCTs, including recurrent HGMCTs, surgically excised from 49 dogs, met this criterion (64% of included dogs). Incomplete surgical margins were significantly associated with local recurrence (odds ratio = 3.97, *p* = 0.037, Table 2), but not with the development of postoperative metastasis or survival time. Of the 19 masses with incomplete surgical margins, 11 (58%) developed local regrowth, while nine (26%) of the 35 masses with complete margins recurred. The median time to local recurrence was 140 days. Seven of the 19 surgical sites with incomplete margins were treated with RT, and 43% (*n* = 3) of these sites experienced MCT regrowth. Data from this study failed to demonstrate a significant local recurrence benefit from concurrent chemotherapy and RT (Table 2).

**Table 2:** Effect of surgical margins and radiation therapy on local recurrence.

Variables	*n*	Local recurrence		*p* value
		*n*	%	
Surgical margins				
Complete	35	9	26	0.037
Incomplete	19	11	58	
Radiation therapy^a^				
No	12	8	67	0.297
Yes	7	3	43	

^aOnly surgical sites with incomplete margins were included in the statistical analysis.^

At the end of the study, 62 dogs were dead or euthanized for the following: MCT-related causes (*n* = 52), undetermined causes (*n* = 6), and causes unrelated to MCT (*n* = 4). Fifteen dogs were censored from the analysis, including 12 that were still alive and three that were lost to follow-up. A subset of the study population had one or more of the following comorbidities: cardiovascular disorders (*n* = 9), neurologic diseases (*n* = 5), musculoskeletal disorders (*n* = 3), melanoma (*n* = 2), hemangiosarcoma (*n* = 1), squamous cell carcinoma (*n* = 1), renal adenocarcinoma (*n* = 1), anaplastic carcinoma (*n* = 1), soft tissue sarcoma (*n* = 1), primary lung histiocytic sarcoma (*n* = 1), duodenal mass (*n* = 1), and chronic renal insufficiency (*n* = 1). No association was found between sex, number of MCTs at presentation, local recurrence, and survival time.

## Discussion

This study reports the outcomes of 77 dogs with HGMCTs treated with various regimens based on clinician and owner discretion. Various studies on canine HGMCTs treated with surgery alone have shown varying survival times, ranging from 98 days to 278 days (16, 19, 33, 34). The MST for all dogs in this study was 317 days, with survival rates at 6-month, 1-year, and 2-year of 69, 50, and 30%, respectively. Our results confirm that dogs with HGMCTs can experience a fair outcome if treated, particularly with surgical intervention. There was a trend toward improved survival time in dogs with complete surgical margins (MST = 882 days) and those receiving concurrent surgery, chemotherapy, and RT (MST = 716 days), emphasizing the importance of local control in dogs with HGMCTs. Hume et al. (15) also reported a survival benefit associated with local tumor control in dogs with grade III MCTs. It should be noted that the number of dogs that did not undergo surgical treatment in our study was small (*n* = 6), and four of these dogs were presented with multiple masses and/or metastasis at initial staging. Combined with the absence of stratification by clinical stage and tumor diameter, these factors likely impacted the strength of our statistical analysis for this subset of the population.

Dogs with LGMCTs can have a good long-term prognosis following complete excision of the primary tumor, making wide surgical excision with adequate margins crucial for a successful outcome (35). Our findings showed that inadequate surgical margins in HGMCTs increased the risk of local tumor regrowth, which occurred in 58% of dogs. Even with complete excision, a significant recurrence risk remained, with 26% of tumors recurring after full resection. This finding is consistent with a previous study, which reported a significantly higher local recurrence rate in HGMCTs (36%) compared to LGMCTs (4%), despite complete resection (35). However, the accuracy of surgical margin assessment in this study may have been compromised by the method used to quantify histologic tumor-free margins. Tangential sectioning, a technique known for its higher sensitivity in identifying incomplete margins (36), was not consistently applied and could have resulted in false-negative classifications. In veterinary oncology, the definition of a complete histologic excision remains undefined, with varying tumor-free margin widths applied inconsistently and often lacking supporting evidence. To address this gap, we adopted the R classification system, widely utilized in human oncology, where a histologic tumor-free margin greater than 0 mm is considered a complete excision and is highly prognostic for most malignant tumors in humans (37).

Theoretically, one would expect local regrowth in all patients with incomplete surgical excision, which was not observed in this study. Several factors have been postulated to explain this, including immune infiltration and eradication of tumor cells post-operatively, the inhibitory effects of anti-invasion factors from connective tissues, the inability of the residual cells to secrete autocrine growth factors to support their survival, and an inadequate follow-up period causing erroneous patient categorization during analyses (38). Interestingly, the local recurrence rate remained high even for completely resected tumors. These recurrent lesions could originate from the surrounding satellite tumor cell populations that were not removed during surgery or from *de novo* tumors near the surgical scars, rather than true local recurrences. In contrast to other literature, we could not find evidence of a negative association between local recurrence and metastasis, as well as survival time (15, 39).

Nodal metastasis was observed in 37% of the patients at initial diagnosis and was associated with a poorer survival outcome, which is comparable to earlier reports (15, 19, 40). Data from this current study surprisingly failed to demonstrate a significant survival advantage of lymphadenectomy in dogs with nodal metastasis. This may be due to the small number of patients with nodal metastasis present at the time of lymphadenectomy and may have affected our statistical analyses. Several studies have shown a favorable therapeutic effect of metastatic lymph node extirpation in dogs with MCTs, and recently, therapeutic lymphadenectomy has gained increased attention in veterinary surgical oncology (15, 30, 41, 42). This study’s lack of an associated survival benefit could be attributed to the non-selective nodal dissection technique, potentially missing metastatic lymph nodes. It has been shown that lymphatic draining pattern of tumors may be aberrant and does not correspond to regional lymph nodes in up to 63% of canine patients due to tumor-induced lymphagiogenesis (43–45). Therefore, non-selective nodal dissection may result in undertreatment and undermine the therapeutic efficacy of lymphadenectomy. Additionally, dogs in this study underwent different staging procedures, meaning that some patients might have been under-staged at the time of initial diagnosis.

It is well established that HGMCTs are highly metastatic. Consequently, the administration of adjuvant chemotherapy has become the standard of care, even when visible metastasis is not observed, to reduce the probability of systemic dissemination of tumor cells (20). Adjuvant systemic therapy did not indefinitely halt disease progression; 79% (*n* = 31/39) of the dogs initially free from metastasis later experienced progression, with 38% (*n* = 15) developing metastatic disease despite chemotherapy. Additionally, 65% of dogs succumbed to their disease despite combination treatment with surgery and chemotherapy, with or without RT. This underscores the need for more effective treatment regimens or strategies for this subset of dogs. Nonetheless, the contribution of adjuvant systemic therapy, with or without RT, to the prolonged survival time should not be disregarded. The MST, 1-year, and 2-year survival rates in this study are longer than those reported in previous studies on dogs that underwent surgical treatment only (16, 19, 33, 34).

The predilection of retrievers and bulldog-related breeds to MCTs observed in this study is consistent with previous reports (46, 47). Mast cell tumors in inguinal and perineal areas have historically been associated with an unfavorable outcome, and recent literature has shown an increased risk of HGMCT development in these locations (32). However, our data indicates that the majority of the HGMCTs were located on the trunk (36%), limb (26%), and head/neck (25%), and we were unable to demonstrate a significant association between these locations and prognosis. Sfiligoi et al. (48) also suggested that dogs with MCTs at the perineal and inguinal areas may not have a worse prognosis. This finding warrants further investigation to determine the prognostic impact of tumor localization, which could help clinicians make more informed treatment decisions.

The retrospective nature of this study presents limitations that should be noted. A primary limitation is the lack of stratification by clinical stage and tumor diameter, potentially introducing bias into the findings. Moore et al. (18) demonstrated that dogs with stage I HGMCT and tumor diameters less than 25 mm can achieve favorable outcomes, underscoring the necessity of such stratification in future analyses. Furthermore, different pathologists and clinicians examined the histopathological slides and patients in this study population, respectively, leading to potential inter-pathologist and clinician variability. Other limitations include the absence of a control group for comparing outcomes between treated and untreated dogs and the small sample size in certain treatment groups. The progression-free interval was not examined as the date of disease progression was not uniformly available in the medical records and some dogs were classified as relapse based on the attending clinician’s clinical judgement without further diagnostic confirmation.

Results from this study suggest that local tumor control and adjuvant medical treatment provide a survival advantage for dogs with HGMCTs compared with findings from other studies that evaluated the outcomes of dogs with HGMCT that received surgical treatment only. Early diagnosis and intervention of HGMCTs are essential as metastasis at initial diagnosis negatively impacts prognosis. Lymphadenectomy did not improve outcomes in this study; however, further investigations into the benefits of sentinel lymph node mapping and biopsy in dogs with HGMCT is recommended. The high rate of local recurrence in completely resected HGMCTs underscores the need for more reliable outcome data to assist surgeons in making informed decisions on resection techniques and improving clinical results. Finally, the failure of adjuvant chemotherapy to impede disease progression remains a significant problem, highlighting the urgent need for better treatment strategies for HGMCTs.

## Data Availability

The original contributions presented in the study are included in the article/supplementary material, further inquiries can be directed to the corresponding authors.
